# Editorial: Immunology of Aging

**DOI:** 10.3389/fimmu.2019.01614

**Published:** 2019-07-10

**Authors:** Graham Pawelec, Sudhir Gupta

**Affiliations:** ^1^Department of Immunology, University of Tübingen, Tübingen, Germany; ^2^Cancer Solutions Program, Health Sciences North Research Institute, Sudbury, ON, Canada; ^3^Division of Basic and Clinical Immunology, University of California, Irvine, Irvine, CA, United States

**Keywords:** immunosenescence, immunogerontology, aging, adaptive immunity, innate immunity

Aging represents a paradox of immunodeficiency and inflammation (inflamm-aging) and autoimmunity (1). Over the lifespan there are changes in the architecture and functioning of the immune system often termed “Immunosenescence.” Recently, there have been major developments in understanding the cellular and molecular bases, and genetic and epigenetic changes, in the innate and the adaptive immune system during aging, and the interactions between these separate arms of vertebrate immunity. The 13 papers in this collection “Immunology of Aging” represent a wide range of investigations by prominent experts in the field, focusing primarily on human aging and disease.

Limited longitudinal studies have begun to reveal biomarkers of immune aging, which may be considered to constitute an “immune risk profile” (IRP) predicting mortality and frailty in the very elderly, as first established in the pioneering Swedish OCTO study (2). Hallmark parameters of the IRP may also be associated with poorer responses to vaccination. The usually asymptomatic infection with the widespread persistent beta-herpesvirus HHV5 (Cytomegalovirus, CMV) has an enormous impact on these immune biomarkers, but according to the circumstances and depending on what is measured, this can translate into a detrimental or a beneficial effect (3). The prevalence of CMV infection in populations in industrialized countries increases with age, and within individuals the degree of immune commitment to anti-CMV responses also increases with age. This may cause pathology by maintaining higher systemic levels of inflammatory mediators (“inflammaging”) and decreasing the “immunological space” available for immune cells with other specificities, or it may exert beneficial “adjuvant-like” effects. Modalities to prevent or reverse immunosenescence may therefore need to include targeting infectious agents such as CMV in a robustly personalized manner.Because of the increasing recognition that CMV has a marked impact on immune parameters commonly associated with age, it is crucial to dissect out whether age or CMV is responsible for altering biomarkers predictive of health status (e.g., frailty) or other important parameters such as response to vaccination (especially seasonal influenza). Hence several of the papers in this collection focus on the effects of CMV on immune parameters.

In their original article, Jackson et al. explore whether T cell responsiveness to a range of CMV proteins is different in younger and older healthy people and whether relaxation of anti-CMV immunosurveillance in later life could contribute to disease. They found that CMV-specific CD4+ T cells secreting the anti-inflammatory cytokine IL 10 were predominantly directed to latency-associated CMV proteins and that these responses were not greater in the elderly than the young. However, the frequency of IFN-γ-secreting CD4+ T cells correlated with latent viral genome copy number in monocytes. They conclude that viremia is rare in the elderly due to the maintenance of T cell responsiveness but that CMV can be an important comorbidity factor in people who are not perfectly healthy and that this could be reflected in the IL 10:IFN-γ ratio of CD4+ anti-CMV T cell responses (Jackson et al.). The potential importance and possible prognostic utility of the IL 10:IFN-γ ratio is also emphasized in the paper by Merani et al. on responsiveness to influenza. This review article summarizes the impact of aging and CMV infection on immune cell function, the response to influenza infection and vaccination, and how the current understanding of aging and CMV can be used to design a more effective influenza vaccine for older adults which will also need to focus on generating appropriate T cell responses, as illustrated in the paper by Jackson et al. Mouse models may also offer insight into how to improve seasonal influenza vaccine responsiveness, as illustrated by Bartley et al. in their consideration of the impact of the gut microbiome and caloric restriction on sublethal influenza infection.

Further complications in analyzing the impact of CMV may arise because most human data are derived from studies using peripheral blood. However, as illustrated by Pangrazzi et al. the bone marrow harbors large amounts of late-stage differentiated CD8 T cells possibly because the production of IL 15 is greater in CMV-infected individuals; this does parallel what is seen in the peripheral blood. Finally, the original article by Hassouneh et al. describes detailed phenotyping results for all peripheral T cells, including CD4+, CD8+, CD4CD8-double negative and NKT cells as well, showing subtle differences between the expression of some surface molecules. For example, expression of the NK-associated receptor CD161 is similar in CMV-seropositive and seronegative young subjects but is different in the elderly, illustrating that CMV effects may be different at different ages. In fact, late-stage differentiated T cells, especially CD8+ T cells (often described in the literature as “senescent”) commonly express surface molecules expressed by (non-T) NK cells. Such receptors include CD85j, which is discussed by Gustafson et al. as an important checkpoint regulator controlling the expansion of CMV-specific CD8+ T cells during aging.

The large accumulations of CMV-specific T cells, also in the bone marrow, may contribute to the state of inflammaging, but it is likely that other immune (and non-immune) cells are also major contributors. Cells of the innate immune system far outnumber those of adaptive immunity and may also be heavily influenced by the presence of CMV, contributing to inflammaging, as discussed by Franceschi et al. in the context of “trained innate immunity”. An evolutionarily more recent persistent virus, HIV, may have some very similar effects, as also discussed in this paper which suggests that the sum total of a person's prior exposures and immunological memory therefor, dubbed the “immunobiography” mostly determines their later-life immune status (Franceschi et al.). The impact of HIV itself is the subject of the original article by Kirk et al. on serum inflammatory mediators in HIV-infected-vs.-non-infected subjects, concluding that chronic infection with HIV, despite its pharmaceutical control, exacerbated the age-associated higher levels of mediators like IL 6 and CRP.

The generally harmful effects of chronic inflammation are not limited to those mediators produced as a result of chronic infection. The article by Frasca and Blomberg starkly illustrates the immune and inflammatory impact of obesity on many health parameters, including decreased and dysregulated B cell function and antibody production as well as inflammaging. Not only does immune dysregulation contribute to inflammaging, but chronicity of inflammatory exposure, as opposed to the necessity of acute inflammation for immune response generation, also negatively influences immune function. Mechanisms whereby this detrimental effect could be mediated are discussed in the review article by Jose et al. focusing on telomere maintenance and telomerase expression. Heightened chronic inflammatory status is also likely to affect innate immune cells as well as the T and B cell effects discussed by Frasca and Blomberg. Much interest in the context of cancer and immunotherapy has recently been raised by studies on so-called myeloid-derived suppressor cells (MDSCs) which may also be relevant in other situations. This is discussed in the context of Alzheimer's Disease by Le Page et al. suggesting that in this instance, unlike in cancer, they may play a positive role. Another type of myeloid cell, essential for the functioning of adaptive immunity in its role as “professional” antigen-presenting cell, may be affected by inflammaging and may itself contribute to this state. Thus, as reviewed by Agrawal et al. dendritic cells (DCs) from older subjects secrete more pro-inflammatory and less anti-inflammatory cytokines and are altered in many other ways, contributing to dysregulated immunity. A further problem related to DC function and antigen presentation relates to the architecture and functionality of the lymph node in elderly subjects, where adaptive immune responses are triggered. As reviewed by Thompson et al. attempts to modulate immunity in the elderly and restore appropriate immune function would need to address this important “checkpoint” too.

The papers collected in this series illustrate only some of the many facets of immune aging, a rapidly developing field now being increasingly recognized as central not only to immune function *per se* in the elderly, but to their general condition of health or frailty.

## Author Contributions

All authors listed have made a substantial, direct and intellectual contribution to the work, and approved it for publication.

### Conflict of Interest Statement

The authors declare that the research was conducted in the absence of any commercial or financial relationships that could be construed as a potential conflict of interest.
